# Integrated global and unique metabolic characteristics to reveal the intervention effect of Yiyi decoction on acute pancreatitis

**DOI:** 10.1371/journal.pone.0310689

**Published:** 2024-11-21

**Authors:** Guanwen Gong, Yongping Wu, Yanwen Jiang, Yuan Cao

**Affiliations:** 1 Department of General Surgery, Affiliated Hospital of Nanjing University of Chinese Medicine, Jiangsu Province Hospital of Chinese Medicine, Nanjing, China; 2 Laboratory of Chemistry, Jiangsu Provincial Institute of Materia Medica, Nanjing, China; 3 Department of Pharmacy, Affiliated Hospital of Nanjing University of Chinese Medicine, Jiangsu Province Hospital of Chinese Medicine, Nanjing, China; Foshan University, CHINA

## Abstract

Yiyi decoction is a Chinese herbal formula for the treatment of acute pancreatitis that has been used in clinical practice for decades. A previous study has suggested that resveratrol, emodin, rhein and their derivatives might be the potential pharmacodynamic components in Yiyi decoction, and researchers have proposed that resveratrol, emodin and rhein are candidate markers for quality control. The present study investigated the intervention effect of Yiyi decoction and its effective components on murine acute pancreatitis using metabolomic approach that integrated global and unique metabolic characteristics. First, serum metabolomics based on the platform of ultra-high performance liquid chromatography coupled with quadrupole time-of-flight mass spectrometry was performed to assess metabolic changes in experimental acute pancreatitis. Second, an in-depth analysis of bile acid metabolism was performed based on an in-house database. Finally, an integrated analysis of the intervention effect of Yiyi decoction and its effective components in response to these metabolic perturbations was performed. As a result, 39 potential biomarkers for the pathogenesis of acute pancreatitis, mainly phospholipids, fatty acids, bile acids and lipoylcarnitines, were screened and annotated. Integrated analysis revealed that the metabolic disorders in acute pancreatitis mice were reversed by Yiyi decoction primarily via regulating glycerophospholipid metabolism, bile acid biosynthesis, carnitine synthesis and fatty acid metabolism. Yiyi decoction components may effectively target the migratory metabolome. Histopathological and biochemical analyses suggested that Yiyi decoction maintained the gut barrier function and inhibited inflammatory cytokines, thus exert anti-acute pancreatitis effects. The present study utilized an approach that integrated global and unique metabolic characteristics to elucidate the underlying mechanisms of Chinese herbal formulas from a metabolomics perspective.

## Introduction

Acute pancreatitis (AP) is a common gastrointestinal condition of the abdomen that leads to hospitalization, it is characterized by premature activation of pancreatic enzymes and local and/or systemic inflammation [1]. The global incidence ranges from 13–45 cases per 100 000 individuals annually, and has continued to increase during the last decade [2]. The typical clinical symptoms of AP include persistent and sharp abdominal pain, accompanied by distension, nausea and vomiting [3]. Approximately 20% of patients with severe AP may develop pancreatic necrosis, sepsis, and multiple organ dysfunction syndrome (MODS), which is the most severe complication in terms of mortality [4]. Specific agents for the treatment of AP are still unavailable despite the substantial burden of this disease [3,5]. Therefore, effective drugs that can combat AP and ameliorate its clinical symptoms are urgently needed.

The therapeutic effects of rhubarb-based Chinese herbal formulas (CHFs) such as Dachengqi decoction and Qingyi decoction have been validated in clinical practice, and recommended in the updated guidelines for the diagnosis and treatment of AP in China (2021) [3]. Yiyi decoction (YYD) is a CHF that has been used to manage AP in clinical practice for decades, in which rhubarb (Rhei Radix Et Rhizoma, RRR) represents a monarch drug and Polygoni Cuspidati Rhizoma et Radix (PCRR), Piperis Kadsurae Caulis and Hirudo serve as adjuvant drugs to improve the effects of rhubarb. Metabolome-oriented network pharmacology research has revealed the potential of resveratrol, emodin, rhein, and their derivatives as efficacious components of YYD, most of which are derived from the RRR monarch drug and the PCRR minister drug [6]. Resveratrol, emodin, and rhein (RER) exhibit multiple beneficial effects, including antimicrobial, anti-inflammatory, and antioxidant effects [7–9], and the anti-AP effects of these components have been well-documented [10–16], suggesting that they are candidate markers for quality control [6]. However, the overall efficacy and underlying mechanism of YYD in AP remain to be clarified. Additionally, the efficacy and mechanism of the combination of resveratrol, emodin, and rhein in a similar ratio found in YYD warrant further verification.

Metabolomics provides a promising analysis methodology to investigate the global metabolic impact after treatment by CHFs. Metabolomics involves an integrated analysis of metabolites in aparticular compartment of an organism and focuses on the physio-pathological conditions of biological systems. Under AP conditions, metabolites involved in different biochemical pathways become dysregulated. Gas chromatography/liquid chromatography-mass spectrometry (GC/LC‒MS) or nuclear magnetic resonance (NMR) based metabolomics studies have been performed using biofluids from AP patients or murine models to analyze global metabolic changes and identify significantly altered metabolites [17–20]. An in-depth understanding of disrupted metabolic profiles will help revealing the potential biomarkers for pathogenesis and uncovering the underlying mechanism, thus contributing to the discovery of specific drugs for AP treatment.

In the present study, a metabolomics strategy integrating global and unique metabolic characteristics was utilized to explore the underlying mechanism of the effects of YYD and RER on AP. First, serum metabolomics was performed to identify the metabolic changes in experimental AP. Second, an in-depth analysis of bile acid metabolism was performed based on an in-house database. Finally, an integrated analysis of the intervention effects of YYD and RER on the metabolic perturbations was performed.

## Methods

### Materials and reagents

Resveratrol, emodin, and rhein were purchased from Chengdu Alfa Biotechnology Co., Ltd, and the purity of each compound was greater than 98%, as determined by HPLC analysis. MS-grade methanol and acetonitrile were obtained from Merck Company Inc. (Darmstadt, Germany). MS-grade formic acid was obtained from Fisher Scientific Company Inc. (Fairlawn, NJ, USA). All other reagents were of analytical grade. Fresh ultrapure water (18.2 MΩ) was prepared using a Milli‐Q water purification system (Millipore, Milford, MA, United States). TNF-α and IL-6 assay kits were purchased from Nanjing Jiancheng Bioengineering Institute (Nanjing, China), while amylase (AMS) was purchased from Nanjing Jin Yibai Biological Technology Co. Ltd. (Nanjing, China). 2-Chloro-l-phenylalanine, which was used as the internal standard (IS), was purchased from J&K Scientific Ltd. (Beijing, China).

### Preparation of YYD

YYD is composed of Rhei Radix et Rhizoma (Lot. 20201203), Polygoni Cuspidati Rhizoma et Radix (Lot. 20201202), Piperis Kadsurae Caulis (Lot. 20201201), and Hirudo (Lot. 20201201) at a ratio of 3:4.5:1:5, and these herbs were purchased from Anhui Wansheng Chinese Herbal Pieces Co., Ltd (Anhui, China). The quality of the above herbs was verified according to the Chinese Pharmacopeia (China Pharmacopoeia Committee, 2020). Briefly, the four herbs were soaked in distilled water for 3 h, and then extracted twice with a 12- fold volume of boiling water for 1 h. The extracted solutions were concentrated under reduced pressure to give a concentration of 1.08 g/mL and then stored at 4°C. UPLC-UV was used to determine the contents of resveratrol, emodin, and rhein in YYD, which indicated contents of 1.20, 2.01, and 1.14 μg/mL respectively.

### Animal experiment

Twenty-eight male SPF C57BL/6 mice (24 ± 1 g) were maintained under controlled temperature (23 ± 2°C), humidity (55 ± 5%) and lighting (12 h light-dark cycles) conditions. After 3 days of acclimatization, the mice were randomly divided into the following four groups: control group (Control), AP group (Model), YYD group (YYD) and active ingredient-treated group (RER) (n = 7 in each group). The AP model was induced by intraperitoneal injection of 8% L-arginine (3.5 g/kg, twice, hourly interval), and the control group was injected with the same volume of sterile normal saline. Then, YYD was intragastrically administered to mice at a dosage of 19.6 g crude drug/kg/d, and RER was intragastrically administered to mice at a dosage of 50 mg/kg/d, which is in accordance with the proportions of the original YYD (resveratrol at 12.5 mg/kg/d, emodin at 25 mg/kg/d, and rhein at 12.5 mg/kg/d). Drugs were administered twice a day with an interval of 8 h. The animal protocol was approved by the Animal Ethics Committee of the Affiliated Hospital of Nanjing University of Chinese Medicine (2018DW-05-06). All efforts were made to minimize animal suffering and the number of animals used for the studies.

After 72 h, the mice were anesthetized by intraperitoneal injection of 1% pentobarbital solution (50 mg/kg), and sacrificed by cervical dislocation under anesthesia. Blood samples were collected, and centrifuged for 10 min at 3000 rpm. The serum was transferred and then stored at -80°C until use. Pancreatic and ileal tissues were collected and fixed in 4% paraformaldehyde solution for histopathological staining.

### Histopathological examination and biochemical analysis

Tissues were embedded in paraffin, stained with hematoxylin and eosin (H&E), and then observed under an optical microscope (Olympus, Japan) at a magnification of 200×. The histopathological scores of pancreatic tissue and ileal tissue were evaluated by two blinded pathologists, using the scoring system reported previously [21, 22], and the data were presented as the mean ± SD (n = 4). The serum levels of TNF-α, IL-6 and AMS were determined according to the instructions of the assay kits.

### Metabolome analysis

#### Serum preparation

Serum (200 μL) was fortified with 600 μL of acetonitrile (containing 20 ng/ml IS) and vortexed for 3 min. After centrifugation at 13000 rpm and 4°C for 10 min, the supernatant was dried under nitrogen gas. Before analysis, the residues were reconstituted in 100 μL of acetonitrile/water (1:1, v/v).

#### Instruments and conditions

Chromatographic separation was performed on an ExionLC system (AB Sciex, Foster City, CA, USA) equipped with a Waters Acquity BEH C18 column (2.1×100 mm, 1.7 μm) at 35°C. In brief, 2 μL of the samples was injected, and a flow rate of 0.3 mL/min was used. Mobile phase A consisted of water with 0.1% formic acid (v/v), and mobile phase B consisted of acetonitrile. The following gradient was utilized: 0–8 min from 5% to 60% mobile phase B, 8–18 min from 60% to 97% mobile phase B, 18–21 min at 97% mobile phase B; and back to the initial ratio of 5% mobile phase B, which was maintained for an additional 4 min for re-equilibration.

A 5600 Q-TOF mass spectrometer (AB Sciex, Foster City, CA, USA) with an electrospray ionization source (Turbo Ionspray) was used for detection under negative and positive ion modes. The parameters of the mass spectrometer were as follows: gas1 and gas2, 45 psi; curtain gas, 35 psi; heat block temperature, 550°C; ion spray voltage, -4.5 kV in negative mode and 5.5 kV in positive mode; declustering potential, 50 V; collision energy, ±35 V; and collision energy spread (CES), ±15 V. A pooled sample, which consisted of a mixture of small aliquots of each sample, was used as the quality control (QC). The QC specimens were analyzed after every five or six samples throughout the entire analysis procedure.

#### Data processing and analytical strategy

All of the raw data files were uploaded using the SCIEX OS software for data extraction. A three-dimensional matrix consisting of sample names (observations), annotated peak indices (RT-m/z pairs), and peak intensities was obtained. The peak intensity was calibrated to that of the internal standard. Variants with an RSD≥40% in QCs were excluded. The pretreated data were imported into SIMCA-P (version 14.1, Umetrics, Umea, Sweden) for OPLS-DA and PLS-DA. Before biomarker filtration and identification, a Pareto-scaled PCA-Class was performed to exclude abnormal samples (out of the 2nd line) and improve the reliability of the data. The results were considered statistically significant at *P* < 0.05. Potential markers were annotated by HMDB (http://www.hmdb.ca/) and LIPID MAPS (https://www.lipidmaps.org/). The results from pathway analysis are presented graphically by MetaboAnalyst 5.0 (http://www.MetaboAnalyst.ca/). Metabolite set enrichment analysis (MSEA) and pathway analysis, which are integrated enrichment analyses and pathway topology analyses, were used to visualize the important metabolic pathways. A small P value and large pathway impact factor revealed that the pathway was significantly affected. The correlation and interaction between biomarkers and the network analysis were established based on the pathway analysis and references.

## Results

### Pathological parameters

**Fig 1:**
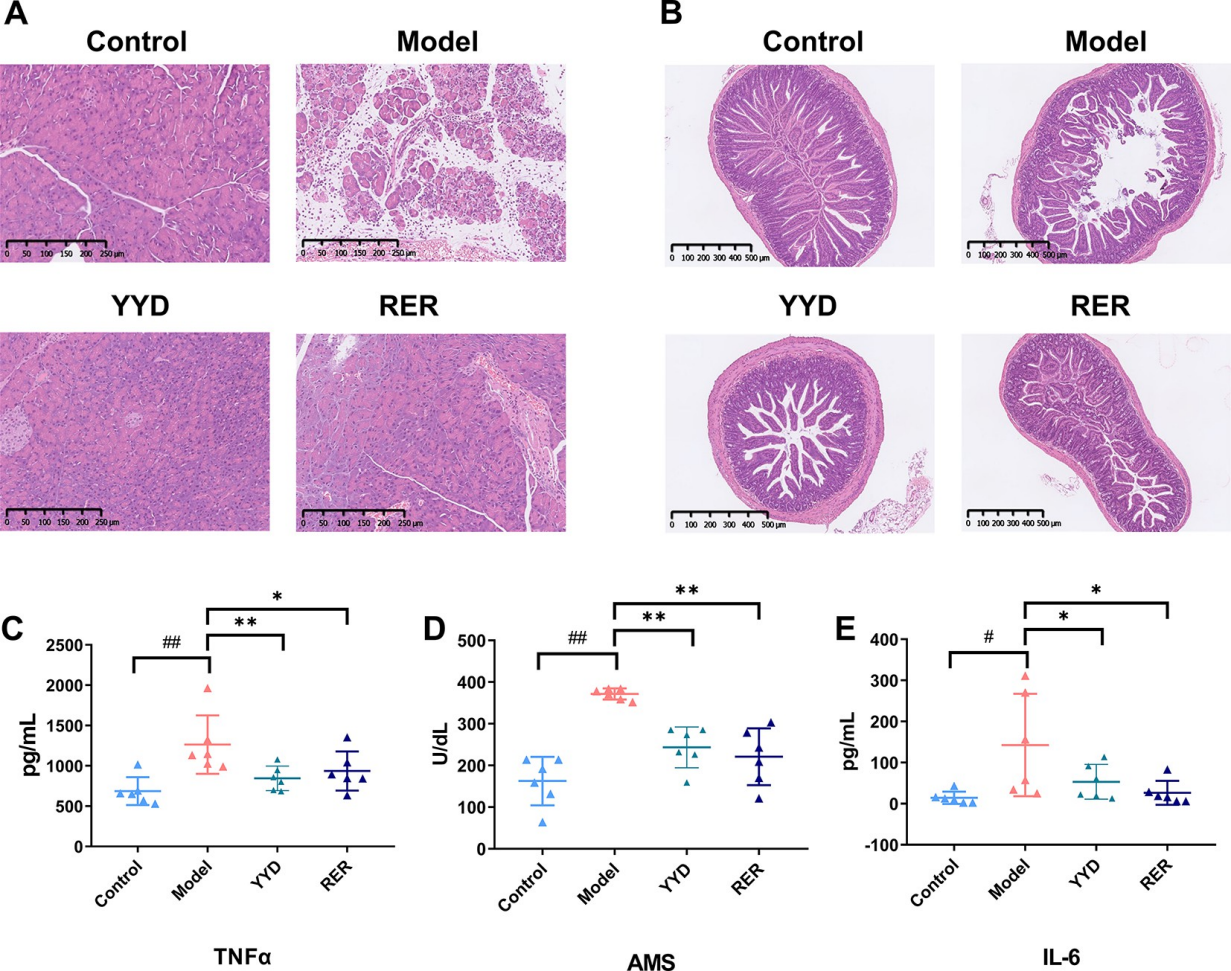
The effects of YYD and RER on 8% L-arginine induced AP in mice. (A) Representative HE-stained histological sections of the ilea. (B) Representative HE-stained histological sections of the pancreas. (C) Serum TNF-alpha level; (D) Serum AMS level; (E) Serum IL-6 level. Data are presented as the mean ± SD (n = 7), *P < 0.05 *vs*. Model, **P < 0.01 *vs*. Model. ^#^ *P* < 0.05 *vs*. Control, ^##^ *P* < 0.01 *vs*. Control.

Histopathological examination revealed that compared with the NS-treated mice and drug-treated mice, the AP model mice had markedly shorter villi and fewer necrotic epithelial cells in the distal ileum (Fig 1A). The ileal histological scores of Control, Model, YYD and RER group were 0, 1.2±0.2, 0.3±0.1, and 1.1±0.2, respectively. Moreover, histiocyte edema, pancreatic cell necrosis, inflammatory cell infiltration, and pancreatic lobule fuzziness, were obviously observed. Conversely, there was mild edema among tissue cells and small inflammatory cell infiltration in the YYD and RER groups (Fig 1B). The pancreatic histological scores of Control, Model, YYD and RER group were 2.2±0.6, 12.5±4.1, 2.5±0.5, and 2.0±0.3, respectively. In addition, abnormal increases in the serum TNF-α, AMS and IL-6 levels (Fig 1C–1E), which are essential for the diagnosis of AP, were detected in the AP model mice. The level of the AMS digestive enzyme was measured to assess the severity of pancreatic injury. A 3-fold increase in the serum AMS level is among the diagnostic criteria for AP, whereas the TNF-α and IL-6 levels are consistently associated with increased severity of AP. Treatment with YYD and RER significantly reversed the increase in the levels of anti-inflammatory cytokines in the AP model mice.

### Data processing

As shown in S1 and S2 Figs (Supplemental information), the overlapping total ion current (TIC) chromatograms of the QC samples demonstrated that acceptable variations occurred during large-scale sample analysis, and all of the samples were in the second line in both positive and negative modes. Therefore, the results were meaningful for further analysis.

### Metabolomic analysis of acute pancreatitis

**Fig 2:**
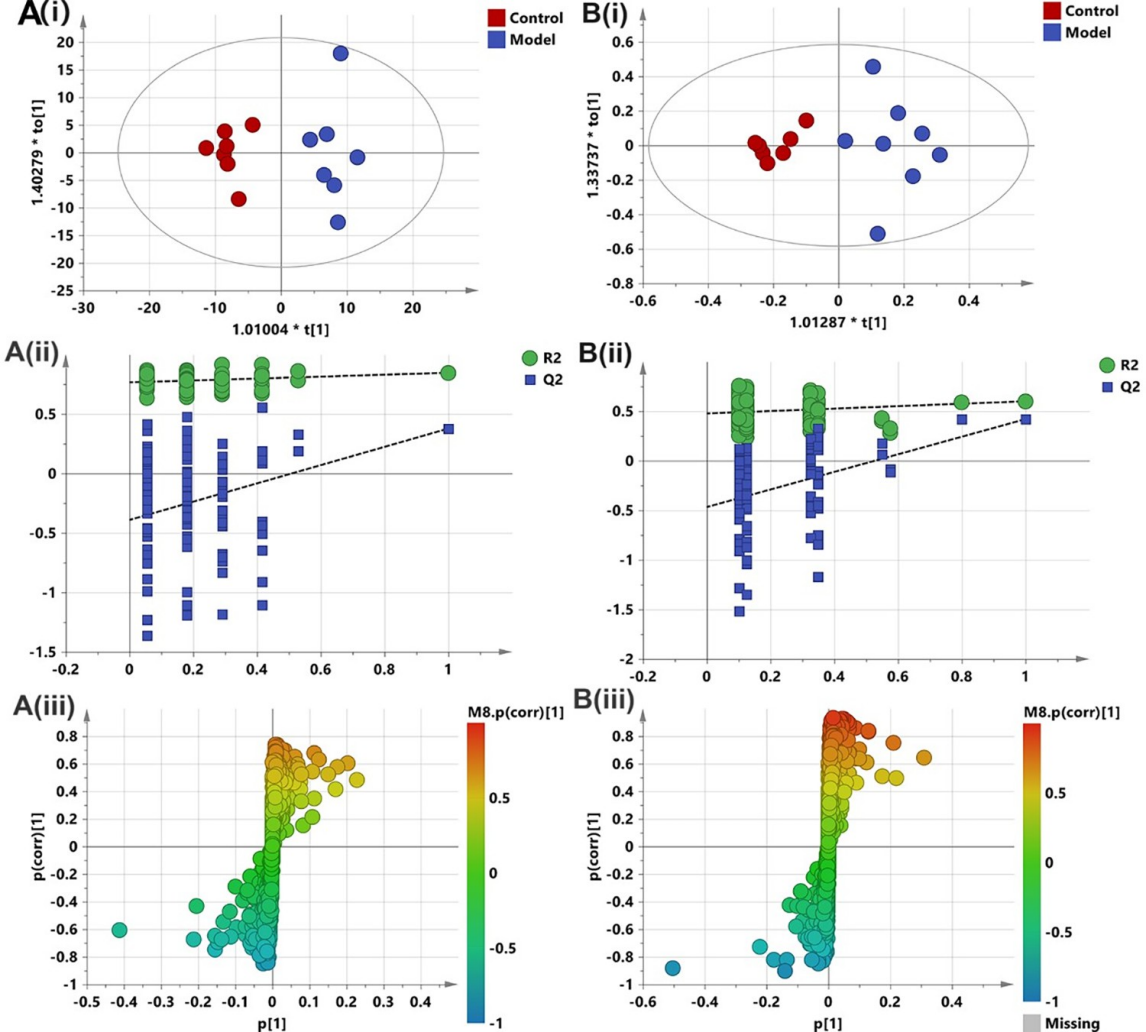
Orthogonal partial least-squares discriminant analysis (OPLS-DA) on the data from ultra-performance liquid chromatography quadrupole/time-of-flight mass spectrometry (UPLC-Q/TOF MS) profiling data from control vs model groups. (A) Negative ion mode (i) Score plot, (ii) Permutation plot (R2X 0.346, R2Y 0.9937, Q2 0.334); (B) Positive ion mode (i) Score plot, (ii) Permutation plot (R2X 0.435, R2Y 0.862, Q2 0.364).

Orthogonal partial least-squares discriminant analysis (OPLS-DA) was utilized to identify different metabolites and screen potential biomarkers, to provide a comprehensive understanding of the AP mechanism. Metabolites with variable importance in the projection (VIP) value >1 and |p(corr)|>0.3 were further subjected to the Mann-Whitney U test to determine the significance of each metabolite, and the *P* < 0.05 was considered to indicate statistical significance. As shown in Fig 2, the control group was clearly distinguished from the model group in the negative mode (Fig 2Ai) and positive mode (Fig 2Bi). Moreover, the permutation test confirmed the satisfactory validity of the OPLS-DA models (Fig 2Aii and 2Bii). Volcano plots (S3 Fig) showing the fold change (FC) and *P* value were generated using RStudio, and the metabolites were annotated. The significantly altered compounds included 14 phospholipids, 8 unsaturated fatty acids, 5 lipoyl-carnitines, 4 bile acids, 4 phenols, 3 organic acids and 1 amino acid (Table 1).

**Table 1:** Identification results of significantly changed metabolites in serum of AP mice.

No.	Name	Formula	Rt	ESI-					ESI+					VIP	*p* corr	*p* value	Fold
				Adduct	m/z	calm/z	ppm	Fragments	Adduct	m/z	calm/z	ppm	Fragments				
1	3-Hydroxyvaleric acid	C_5_H_10_O_3_	2.84	M-H	117.0559	117.0557	1.54	91	-	-	-	-	-	4.74	0.68	0.0243	1.44
2	Pyrocatechol sulfate	C_6_H_6_O_5_S	2.98	M-H	188.9863	188.9863	-0.11	188, 109, 108, 79						3.49	0.47	0.0243	1.73
3	Phenol sulphate	C_6_H_6_O_4_S	3.26	M-H	172.9919	172.9914	2.89	172, 93, 79, 65	-	-	-	-	-	16.77	-0.61	0.0193	0.60
4	Phenylglucuronide	C_12_H_14_O_7_	3.41	M-H	269.0668	269.0667	0.45	175, 113, 93, 85, 59	-	-	-	-	-	1.62	-0.60	0.0380	0.56
5	Hippuric acid	C_9_H_9_NO_3_	3.61	M-H	178.0513	178.0510	1.85	178, 134, 77	M+H	180.0655	180.0655	-0.11	105, 77	2.33	0.58	0.0380	2.06
6	Hydroxyferulic acid	C_10_H_10_O_5_	4.13	M-H	209.0460	209.0456	2.15	209, 165, 121	M+H	211.0603	211.0601	0.95	149, 121, 65	2.86	0.52	0.0380	1.34
7	Equol 4’-sulfate	C_15_H_14_O_6_S	5.39	M-H	321.0441	321.0438	0.84	321, 241, 135, 121, 119, 79						4.41	0.54	0.0193	1.63
8	N-Heptanoylglycine	C_9_H_17_NO_3_	5.39	M-H	186.1136	186.1136	0.16	74	M+H	188.128	188.1281	-0.53	95, 67	1.03	-0.51	0.0380	0.65
9	3-Oxocholic acid	C_24_H_38_O_5_	7.90	M+FA-H	451.2696	451.2701	-1.17	451, 423, 405, 387, 353	M+H	407.2794	407.2792	0.49	407, 389, 371, 353, 335, 159, 145, 135	5.42	-0.54	0.0305	0.48
10	Ursocholic acid	C_24_H_40_O_5_	8.01	M+FA-H	453.2849	453.2858	-1.94	407, 345, 343, 289, 233	-	-	-	-	-	2.93	-0.58	0.0469	0.43
11	Ursodeoxycholic acid	C_24_H_40_O_4_	8.26	M+FA-H	437.2899	437.2909	-2.20	391, 195	-	-	-	-	-	1.51	-0.40	0.0380	0.59
12	Dodecanoylcarnitine	C_19_H_37_NO_4_	8.89	-	-	-	-	-	M+H	344.2800	344.2795	1.34	344, 285, 183, 144, 85	1.09	-0.56	0.0031	0.58
13	LysoPC(14:0/0:0)	C_22_H_46_NO_7_P	9.68	M+FA-H	512.2989	512.2994	-0.98	512, 452, 227	M+H	468.3082	468.3085	-0.58	468, 450, 285, 184, 104	2.23	-0.71	0.0021	0.78
14	Deoxycholic acid	C_24_H_40_O_4_	9.77	M+FA-H	437.2899	437.2909	-2.20	391, 355, 347, 345, 329, 327	-	-	-	-	-	1.94	-0.59	0.0305	0.45
15	LysoPC(20:5/0:0)	C_28_H_48_NO_7_P	9.81	M+FA-H	586.3157	586.3150	1.13	586, 526, 301, 257, 224	M+H	542.3240	542.3241	-0.18	542, 524, 258, 184, 104	4.74	-0.65	0.0092	0.70
16	LysoPC(16:1/0:0)	C_24_H_48_NO_7_P	10.06	M+FA-H	538.3133	538.3150	-3.23	538, 478, 253, 224	M+H	494.3242	494.3241	0.16	494, 476, 311, 258, 184	6.38	-0.65	0.0243	0.62
17	Tetradecanoylcarnitine	C_21_H_41_NO_4_	10.24	-	-	-	-	-	M+H	372.3105	372.3108	-0.91	372, 313, 211, 144, 85	2.37	-0.71	0.0014	0.62
18	Hydroxyhexadecanoic acid	C_16_H_32_O_3_	10.41	M-H	271.2279	271.2279	0.11	271, 211	-	-	-	-	-	1.13	-0.68	0.0071	0.63
19	LysoPE(22:6/0:0)	C_27_H_44_NO_7_P	10.53	M-H	524.2778	524.2783	-0.95	524, 327, 283, 229, 214, 196, 140	M+H	526.2928	526.2928	-0.04	526, 508, 385, 354, 311	3.59	0.46	0.0343	1.17
20	LysoPE(20:4/0:0)	C_25_H_44_NO_7_P	10.62	M-H	500.2775	500.2783	-1.60	500, 303, 259, 196, 140	M+H	502.2923	502.2928	-1.04	502, 484, 361, 330, 287, 269	5.20	0.84	0.0009	1.47
21	Hexadecenoylcarnitine	C_23_H_43_NO_4_	10.62	-	-	-	-	-	M+H	398.3265	398.3265	0.03	398, 339, 237, 144, 85	1.72	-0.63	0.0009	0.41
22	11-HEPE	C_20_H_30_O_3_	10.76	M-H	317.2121	317.2122	-0.38	317, 299, 255, 179, 135, 107	-	-	-	-	-	6.32	-0.75	0.0092	0.42
23	LysoPI(18:2/0:0)	C_27_H_49_O_12_P	10.76	M-H	595.2860	595.2889	-4.85	595, 415, 315, 279, 241, 152	-	-	-	-	-	1.84	-0.67	0.0092	0.67
24	LysoPI(20:4/0:0)	C_29_H_49_O_12_P	10.80	M-H	619.2872	619.2889	-2.73	619, 439, 315, 303, 241, 152	-	-	-	-	-	5.92	-0.67	0.0071	0.73
25	LysoPC(16:0/0:0)	C_24_H_50_NO_7_P	11.48	M+FA-H	540.3302	540.3307	-0.93	540, 480, 255, 242, 224	2M+H	991.6721	991.6723	-0.16	599, 478, 184, 86	8.73	-0.73	0.0044	0.84
26	10-HDoHE	C_22_H_32_O_3_	11.55	M-H	343.2272	343.2279	-1.95	343, 325, 281, 234, 205, 161, 133, 107	-	-	-	-	-	5.99	-0.69	0.0305	0.54
27	LysoPI(20:3/0:0)	C_29_H_51_O_12_P	11.64	M-H	621.3053	621.3045	1.22	621, 441, 315, 305, 241, 152	-	-	-	-	-	1.04	-0.66	0.0152	0.55
28	11-HETE	C_20_H_32_O_3_	11.73	M-H	319.2281	319.2279	0.72	319, 301, 257, 179, 163, 135	-	-	-	-	-	8.78	-0.67	0.0469	0.66
29	Palmitoylcarnitine	C_23_H_45_NO_4_	11.87	-	-	-	-	-	M+H	400.3411	400.3421	-2.60	400, 341, 239, 144, 85	2.87	-0.59	0.0044	0.61
30	LysoPC(18:1/0:0)	C_26_H_52_NO_7_P	11.93	M+FA-H	566.346	566.3463	-0.53	566, 506, 281, 242, 224	M+H	522.3550	522.3554	-0.80	504, 339, 258, 184, 166, 124, 104, 86	20.04	-0.88	0.0205	0.62
31	LysoPE(18:1)/0:0)	C_23_H_46_NO_7_P	11.93	M-H	478.293	478.2939	-1.88	478, 281, 196	M+H	480.3088	480.3085	0.69	480, 462, 419, 339, 308	2.61	0.68	0.0021	2.35
32	LysoPG(18:2/0:0)	C_24_H_45_O_9_P	11.99	M-H	507.2704	507.2728	-4.81	507, 282, 279, 224, 152	-	-	-	-	-	1.24	-0.62	0.0118	0.68
33	Oleoylcarnitine	C_25_H_47_NO_4_	12.18	-	-	-	-	-	M+H	426.3574	426.3578	-0.91	426, 367, 144, 85	3.00	-0.52	0.0343	0.68
34	Stearidonic acid	C_18_H_28_O_2_	13.43	M-H	275.2014	275.2017	-0.91	275, 231	M+H	277.2167	277.2162	1.80	277, 235, 185, 163, 149, 121, 93, 79	1.49	-0.66	0.0193	0.60
35	LysoPC(20:1/0:0)	C_28_H_56_NO_7_P	14.06	M+FA-H	594.3772	594.3776	-0.67	594, 534, 309, 224	M+H	550.3864	550.3867	-0.58	550, 532, 184, 104	2.32	0.46	0.0021	1.51
36	PC 25:1; O	C_33_H_64_NO_9_P	14.66	-	-	-	-	-	M+H	650.4385	650.4392	-1.00	650, 184	1.48	-0.54	0.0266	0.59
37	Neuroprotectin D1/Resolvin D5	C_22_H_32_O_4_	15.19	M-H	359.2222	359.2228	-1.61	359, 341, 327, 315, 297	-	-	-	-	-	1.17	0.70	0.0041	1.53
38	Arachidonic acid	C_20_H_32_O_2_	15.60	M-H	303.2337	303.2330	2.47	303, 259	-	-	-	-	-	5.75	0.63	0.0193	1.46
39	Oleic acid	C_18_H_34_O_2_	17.49	M-H	281.2488	281.2486	0.71	281, 219	-	-	-	-	-	9.59	0.60	0.0071	1.53

### Metabolomic analysis of the effects of YYD and RER on acute pancreatitis

**Fig 3:**
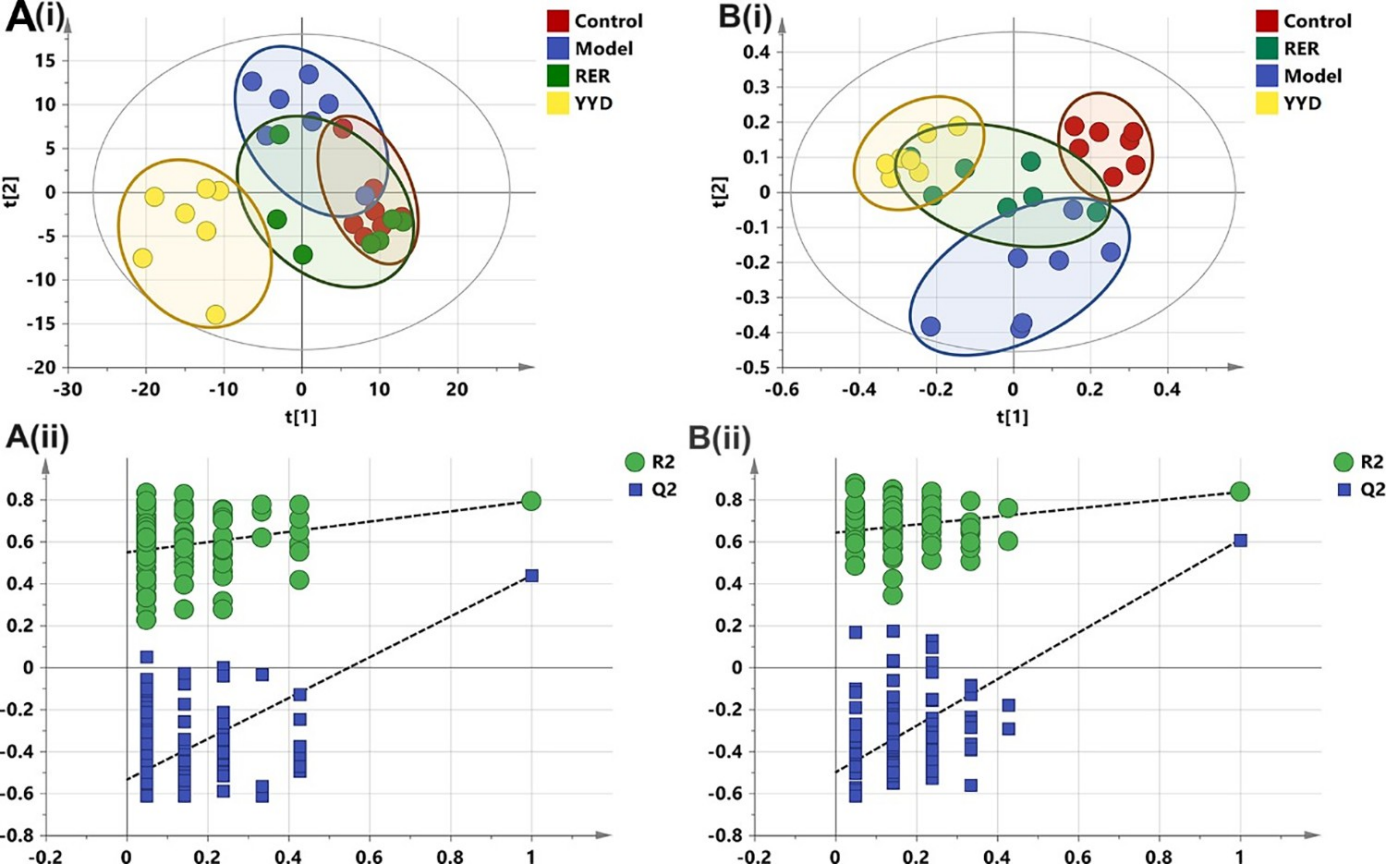
Partial least squares discriminate analysis (PLS-DA) analysis and permutation plot between the control, model, YYD and RER groups. (A) Negative ion mode (i) Score plot, (ii) Permutation plot (R2X 0.634, R2Y 0.829, Q2 0.500); (B) Positive ion mode (i) Score plot, (ii) Permutation plot (R2X 0.603, R2Y 0.887, Q2 0.683).

Partial least-squares discriminant analysis (PLS-DA) was also performed to observe the distances among the groups. As shown in Fig 3, the model and control groups were differentiated in both positive and negative modes. The RER group was closer to the control group than to the YYD group, which indicated that RER might be more effective at regulating the migratory metabolome. The YYD points were scattered far from the other groups, which may have been due to the detection of xenobiotics in the YYD group.

**Fig 4:**
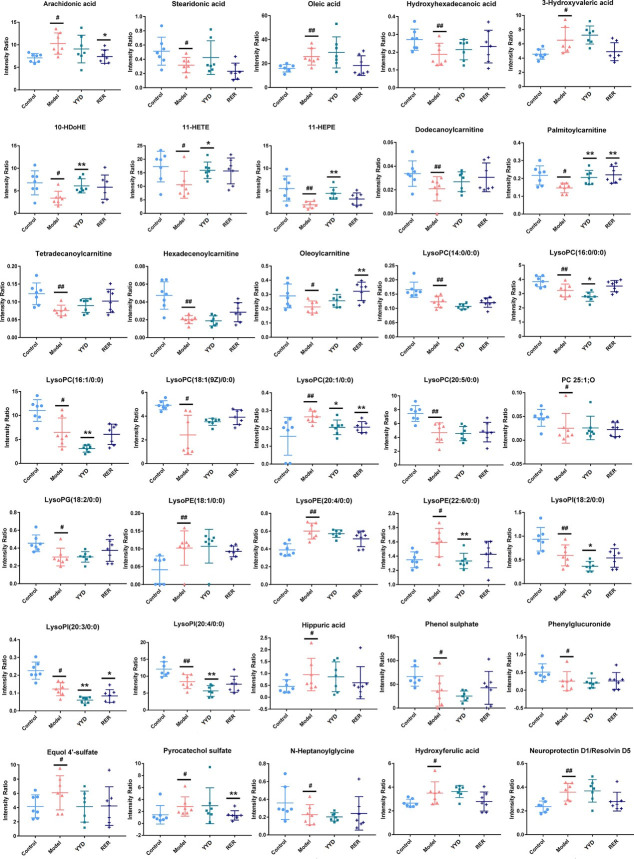
Intensity comparation of significantly changed metabolites of four groups of control, model, YYD and RER groups. ^#^ *P* < 0.05 compared with Control; ^##^ *P* < 0.01 compared with Control; * *P* < 0.05 compared with Model; ** *P* < 0.01 compared with Model.

The intragroup comparisons of individual metabolites are presented in histograms (Fig 4). Phospholipids, fatty acids (FAs), lipoylcarnitines and bile acids (BAs) were disrupted, with decreased FA metabolites and lipoylcarnitines after modeling. The changes in the abovementioned metabolites were reversed after treatment with YYD and RER. Further, RER effectively reversed more metabolites than YYD.

### Bile acid metabolic analysis

**Fig 5:**
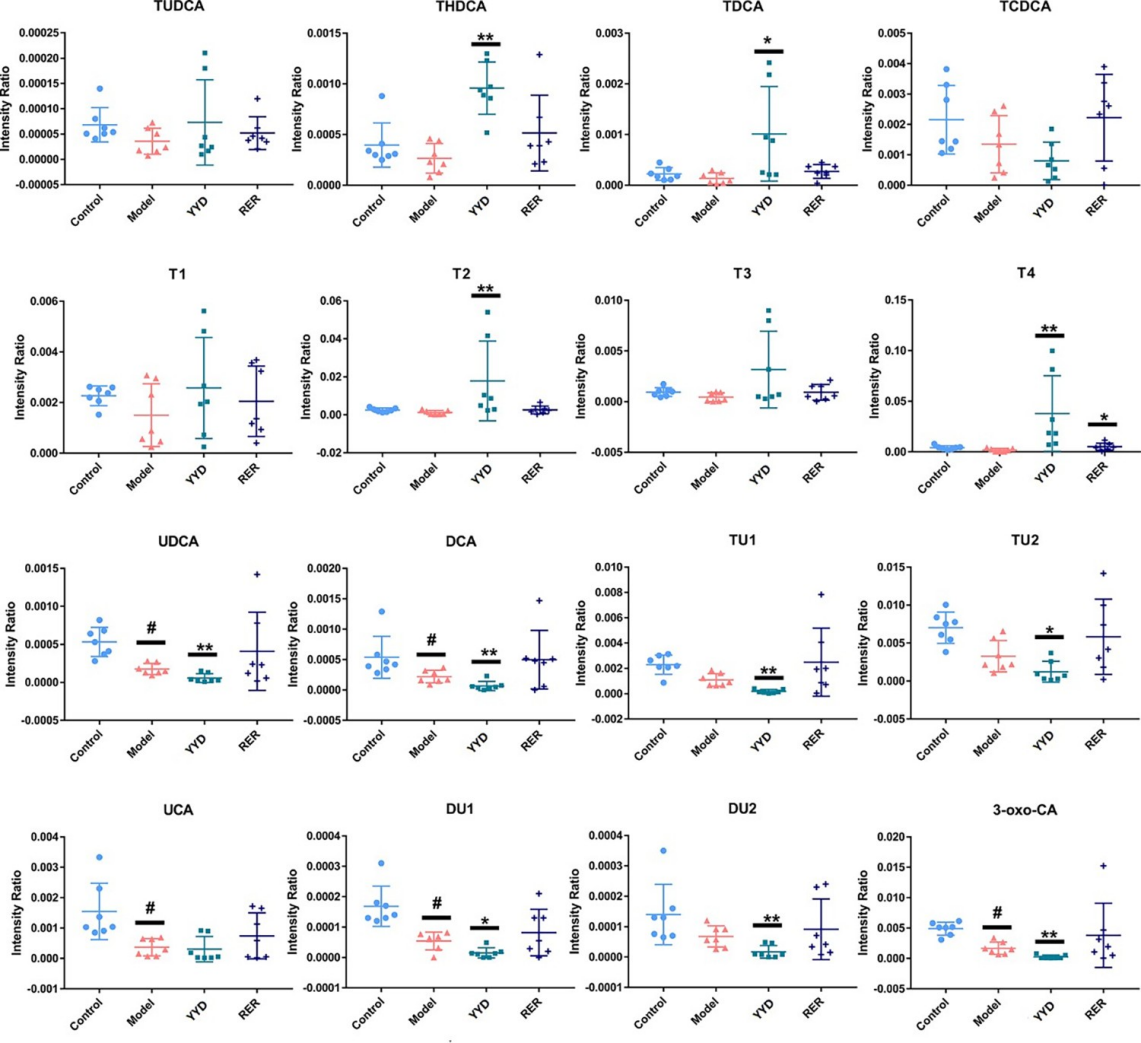
Intensity comparation of significantly changed bile acids of four groups. ^#^ *P* < 0.05 compared with Control; ^##^ *P* < 0.01 compared with Control; * *P*<0.05 compared with Model; ** *P* < 0.01 compared with group Model.

BAs are important host-derived and microbially modified signaling molecules that are synthesized in the liver. The concentrations of BAs are important indicators of either the pathological or physiological status of certain organs, particularly the liver and intestine. Because disorders of BA homeostasis are implicated in a variety of diseases including pancreatitis, the present study further analyzed the BAs. A total of 16 BAs were extracted from the serum (Table 2, S4 Fig), and their intensities were compared via a histogram (Fig 5). Almost all of the BAs showed a downward trend after modeling, with only five BAs (DCA, UDCA, 3-oxo-CA, UCA, and DU1) exhibiting statistical significance. Although none of the BAs were significantly reversed in the dosed group, a tendency toward improvement was observed, especially for THDCA, T2, T4, and TDCA in the YYD group and for TCDCA, DCA, TU2, TU1, UDCA, 3-oxo-CA, DU2, UCA, and DU1 in the RER group.

**Table 2:** Identification results of significantly changed bile acids in the serum of AP mice.

Name	Formula	Rt min	Adduct	calm/z	m/z	ppm	Fragments	*p* value		
								Control *vs* Model	Model *vs* YYD	Model *vs* RER
TUDCA	C_26_H_45_NO_6_S	5.95	M-H	498.2895	498.2910	3.01	498, 80	0.6058	0.8665	0.6058
THDCA	C_26_H_45_NO_6_S	6.37	M-H	498.2895	498.2900	1.00	498, 80	0.743	0.0059	0.2359
TDCA	C_26_H_45_NO_6_S	7.22	M-H	498.2895	498.2879	-3.21	498, 80	0.673	0.0289	0.1672
TCDCA	C_26_H_45_NO_6_S	7.53	M-H	498.2895	498.2889	-1.20	498, 80	0.673	0.1893	0.1672
T01	C_26_H_45_NO_7_S	5.51	M-H	514.2844	514.2855	2.14	514, 80	1	0.6943	0.3704
T02	C_26_H_45_NO_7_S	5.66	M-H	514.2844	514.2853	1.75	514, 80	0.4234	0.0059	0.3704
T03	C_26_H_45_NO_7_S	6.3 3	M-H	514.2844	514.2835	-1.75	514, 80	0.4807	0.1893	0.4234
T04	C_26_H_45_NO_7_S	6.4	M-H	514.2844	514.2859	2.92	514, 80	0.3213	0.0006	0.0464
UDCA	C_24_H_40_O_4_	8.26	M+FA-H	437.2909	437.29	-2.06	437, 391	0.036	0.0037	0.9626
DCA	C_24_H_40_O_4_	9.79	M+FA-H	437.2909	437.2913	0.91	437, 391	0.0464	0.0037	0.4807
TU1	C_24_H_40_O_5_	7.05	M+FA-H	453.2858	453.2867	1.99	453, 407	0.1388	0.0003	0.4234
TU2	C_24_H_40_O_5_	7.4	M+FA-H	453.2858	453.2867	1.99	453, 407	0.1388	0.0289	0.8884
UCA	C_24_H_40_O_5_	8.05	M+FA-H	453.2858	453.2875	3.75	453, 407	0.0274	0.2319	0.743
DU1	C_24_H_38_O_5_	7.36	M+FA-H	451.2701	451.2684	-3.77	451, 405	0.0464	0.014	0.4807
DU2	C_24_H_38_O_5_	7.65	M+FA-H	451.2701	451.2699	-0.44	451, 405	0.3213	0.0037	0.3704
3-oxo-CA	C_24_H_38_O_5_	7.93	M+FA-H	451.2701	451.2688	-2.88	451, 405	0.036	0.0012	0.8884

### Pathway analysis

**Fig 6:**
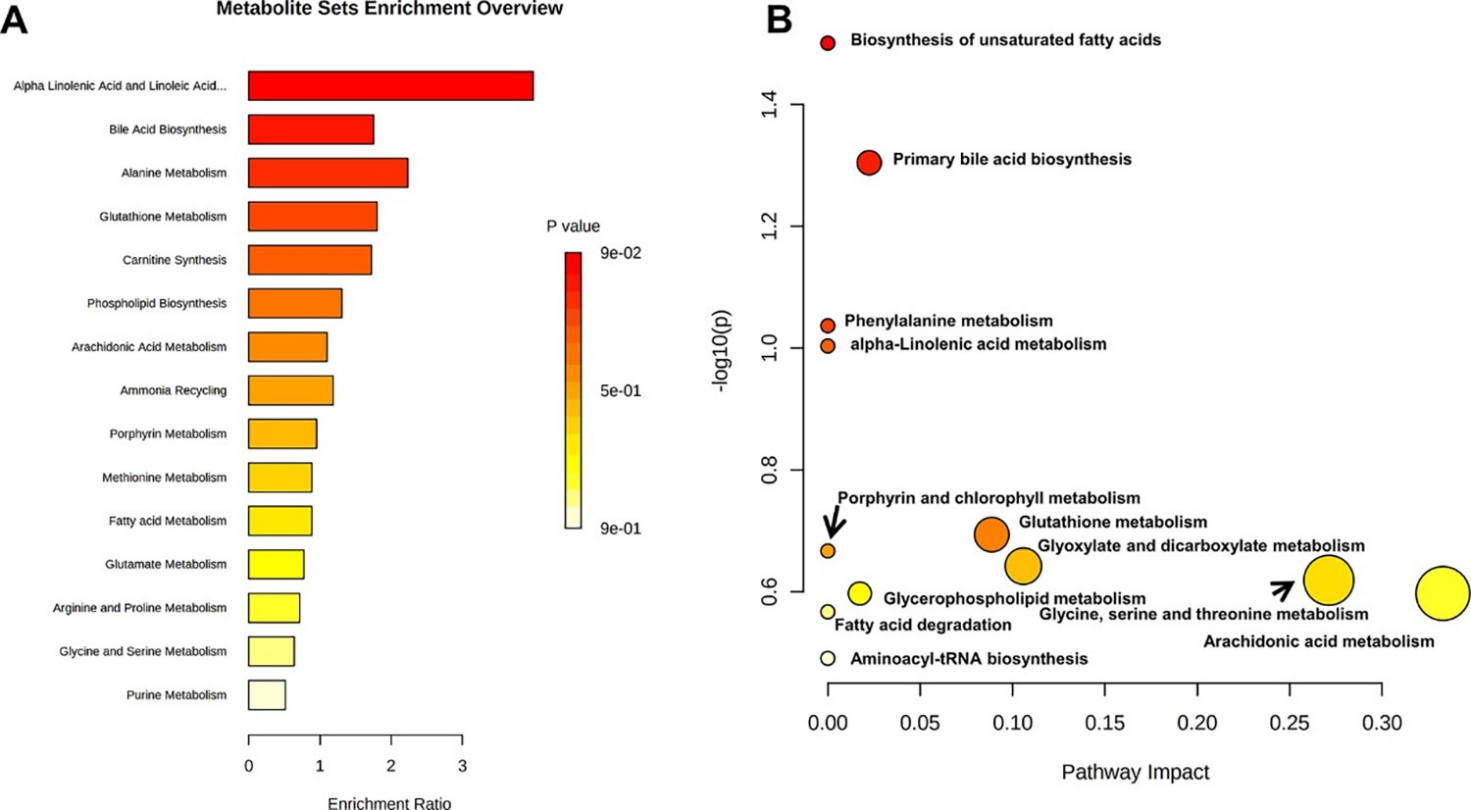
Summary of metabolic pathways significantly enriched in YYD/RER treatment. (A) Enrichment overview of the top 15 metabolic pathways associated with YYD/RER treatment-attributable metabolites. (B) Bubble plot of metabolic pathways identified by MESA analysis of YYD/RER treatment-attributable metabolites identified metabolic pathways.

Pathway analysis revealed that the underlying regulatory effects of YYD and RER on AP in mice were mainly attributed to lipid metabolism, energy metabolism and amino acid metabolism. The important metabolic pathways included glycerophospholipid metabolism, BA biosynthesis, AA metabolism, UFA biosynthesis, FA oxidation, FA degradation, and phenylalanine metabolism (Fig 6).

## Discussion

Our previous study revealed the potential efficacious materials and candidate quality markers for YYD treatment. In this study, we further investigated the effect of YYD and its effective components, namely, RER, on AP using a metabolomic approach that integrated global and unique metabolic characteristics.

AP is an inflammatory disease. During AP, a waterfall-like cascade release of proinflammatory cytokines, such as TNF-α and IL-6, leads to pancreatic inflammation and necrosis. The constantly activated inflammatory response evolves into MODS and systemic inflammatory response syndrome (SIRS). Both YYD and RER exerted anti-inflammatory effects by significantly reversing the abnormal increase in the serum TNF-α and IL-6 levels in AP mice. The therapeutic effects of these compounds on AP were further confirmed by H&E staining of pancreatic and ileal sections from the respective groups. As expected, pancreatic edema, hemorrhage, necrosis, and inflammation were alleviated by treatment with YYD and RER. Previous studies have confirmed the therapeutic effect of resveratrol, emodin and rhein against AP due to their anti-inflammatory and antioxidant properties in experimental AP [11, 12, 16]. The present study demonstrated that the RER combination at levels similar to those found in YYD provided an effective treatment for AP, partly *via* the inhibition of proinflammatory cytokine release.

Accumulating clinical and experimental evidence has shown that homeostatic metabolism of lipids and fatty acids (FAs) is commonly disrupted during AP [23]. The significantly altered metabolites in the serum of the L-arginine murine model included elevated levels of unsaturated fatty acids (UFAs) and *O*-phosphocholine, as well as reduced levels of lysophospholipids, lipoylcarnitines and bile acids (BAs). Alterations in the levels of phospholipids, which are crucial components of cell membranes, might reflect the decomposition of tissues during AP progression [24], which is consistent with the histopathological observations. Moreover, glycerophospholipids have been reported to play important roles in membrane-dependent processes involved in the TLR response [25]. Thus, the dysregulation of glycerophospholipid metabolism influences the secretion of cytokines, such as TNF-α and IL-6, thereby promoting inflammation.

In general, the degradation of phospholipids generates one or two FAs. FAs have an impact on cell and tissue functions as energy sources and membrane components [19]. Since Because FAs are reportedly involved in inflammatory pathology and disturbed FA metabolism easily leads to hyperlipidemia, FAs are inevitably implicated in AP progression [26]. For example, oleic acid, linoleic acid, and arachidonic acid (AA) induce acinar cell injury at high concentrations, thereby resulting in pancreatitis [27]. Substantially elevated serum FA levels have been observed in the AP population, and abnormal fluctuations in FA levels (such as those of AA and linoleic acid) have been reported to be correlated with the deterioration of AP [28]. A meta-analysis including 20 reports from 11 countries has revealed that UFAs derived from the lipolysis of unsaturated visceral triglycerides increased systemic injury and organ failure during pancreatitis [29]. In the present study, an increase in AA was observed. As the precursor of prostaglandins, an increase in AA might induce inflammation [30, 31]. Moreover, changes in lysophospholipid and UFA levels were reversed after administration of YYD and RER, indicating that YYD and RER inhibit the inflammatory response to alleviate AP symptoms.

Carnitine plays an important role in FA β-oxidation (FAO), which transfers long-chain fatty acids (LCFAs) across the mitochondrial membrane in the form of lipoylcarnitine. Decreased lipoylcarnitine levels usually indicate impaired FAO, which can result in lipid accumulation, lipid peroxidation, oxidative stress, irreversible mitochondrial damage, and even immune dysfunction [32, 33]. Moreover, lipid accumulation induced ROS promote the secretion of cytokines via NF-κB. In the present study, lipoylcarnitines were expressed at lower levels in the AP group, but both YYD and RER treatment reversed these changes, especially for oleoylcarnitine and palmitoylcarnitine. These results suggested that YYD and RER may improve AP through FA oxidation regulation.

BAs are involved in the pathogenesis of pancreatitis due to their role in the induction of cytosolic calcium overload and subsequent cell necrosis [34–36]. Disordered BA metabolism may cause cholestasis or lead to the reflux of BAs into the pancreas, which is an inducer of AP [37, 38]. In the murine model, almost 16 detected BAs showed a decreasing trend, and an increasing trend in these BAs was observed after treatment with YYD and RER. Among them, UDCA and TCDCA have protective effects in acute biliary pancreatitis, and UDCA has been approved for use in gallstone dissolution and in treating primary biliary cholangitis [39, 40].

**Fig 7:**
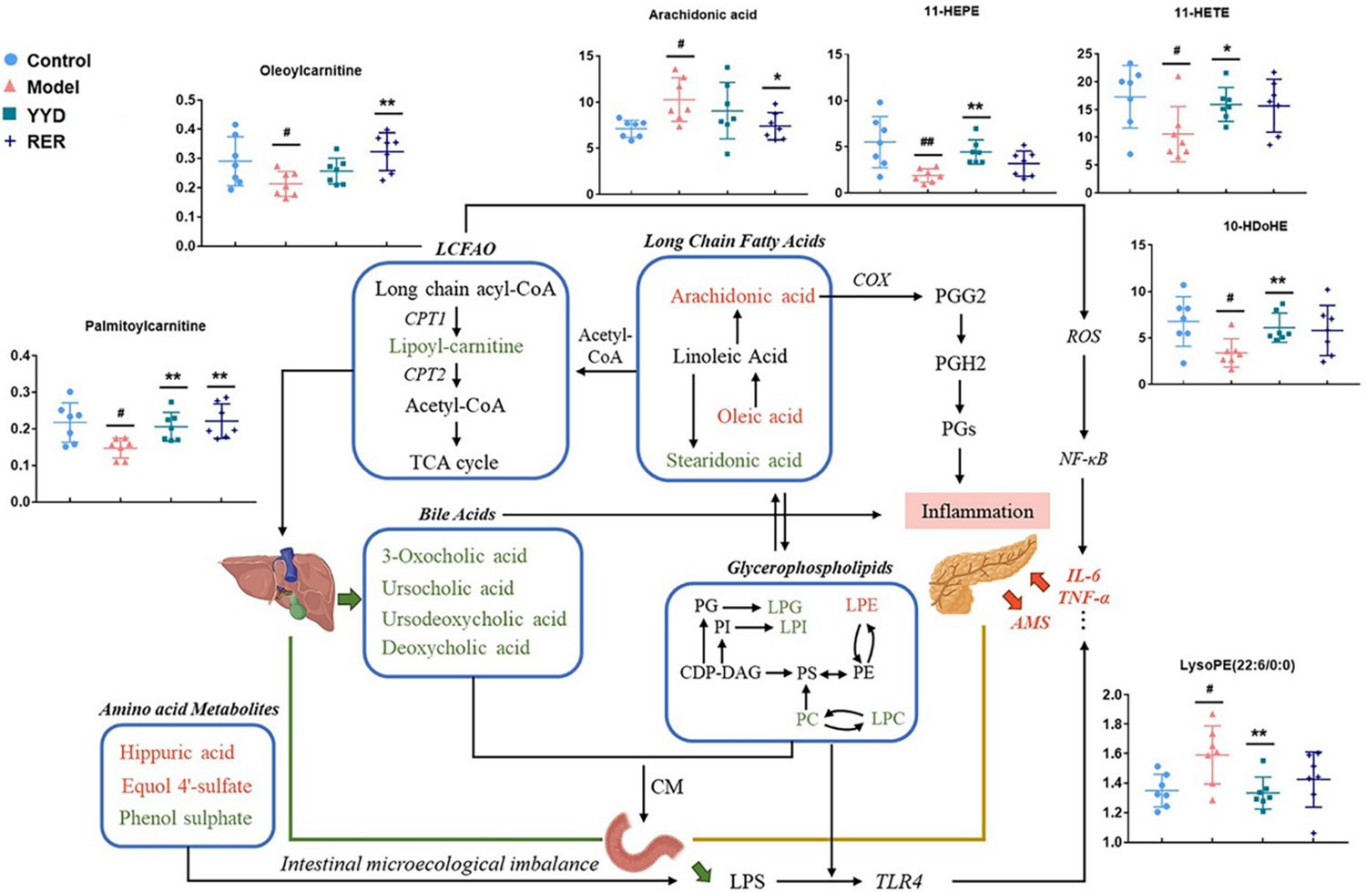
Altered metabolites and pathways implicated in AP that could be ameliorated by both treatment of YYD and RER in murine plasma. Metabolites colored in green were decreased in AP model while red were increased. Reversed metabolites were illustrated with histogram. LCFAO: long chain fatty acid oxidation; CPT1: carnitine-palmitoyltransferase-1; CPT1: carnitine-palmitoyltransferase-2; TCA cycle: tricarboxylic acid cycle; COX: cyclooxyganese; PGG2: Prostaglandin G2; PGH2: Prostaglandin H2; PGs: Prostaglandins; CM: chylomicron; LPS: lipopolysaccharide; NF-κB: nuclear factor kappa-B; TLR4: Toll-like receptor 4.

BAs undergo enterohepatic circulation [41]. The liver and pancreas communicate with the intestine through bile ducts and pancreatic ducts. BA metabolism is an important part of lipid metabolism. BAs can form chylomicrons (CMs) together with phospholipids in the intestine, which is an important metabolic pathway for cholesterol and triglycerides. Disordered BA metabolism always implies liver damage, and dyshomeostasis of the gut-liver-pancreas axis may influence host-microbe interactions [42]. The decreased villus length and necrotic epithelial cells observed in histologic sections of the distal ileum implied a damaged intestinal barrier. Subsequently, toxins, such as lipopolysaccharides (LPSs), produced by microbes can penetrate into systemic circulation more easily. LPS activates Toll-like receptor 4 (TLR4), a key transmembrane recognition receptor, to release IL-6, TNF-α and other proinflammatory factors, thus promoting inflammation [43]. The 16 BAs detected in the present study included both primary and microbially modified secondary BAs, implying a defective intestinal barrier. Rhein, emodin, and resveratrol have been reported to ameliorate SAP-induced intestinal barrier injury and ameliorate AP-associated liver damage [11–16]. In YYD, rhein, emodin, resveratrol and their derivatives have been found to be widely distributed in the bile, intestine and liver. In addition, microbe-derived aromatic substances, such as hippuric aid, equol 4’-sulfate, and phenol sulfate, showed obvious fluctuations in the model group compared to the control group, further confirming the disruption of intestinal homeostasis in AP [44]. After treatment, the aberrant shift was recovered, especially after RER treatment. Taken together, these results indicated that YYD and RER might achieve anti-AP effects by regulating disordered lipid and BA metabolism (Fig 7). However, further experiments are essential to reveal the underlying molecular mechanism involved.

## Conclusion

The present study utilized strategy that integrates global and unique metabolic characteristics to reveal the efficacy and mechanism of YYD and RER on murine AP. Because RER can be easily extracted, prepared, and subjected to strict quality control, this combination holds potential for further development as a lead drug in the treatment of AP.

## Supporting information

S1 Fig The overlapping total ion chromatograms (TICs) of QC samples in positive and negative modes. (DOCX)

S2 Fig PCA-Class score plots in positive and negative modes. (DOCX)

S3 Fig Volcano plots of control *vs* model. (DOCX)

S4 Fig Structures of 16 bile acids extracted in the serum of AP mice. (DOCX)

## Abbreviations

AMS: amylase
AP: acute pancreatitis
BA: bile acid
CA: cholic acid
CHFs: Chinese herbal formulas
DCA: deoxycholic acid
DU: dihydroxycholic acid
OPLS-DA: orthogonal partial least-squares discriminant analysis
PCA: principal component analysis
PCRR: Polygoni Cuspidati Rhizoma et Radix
PLS-DA: partial least-squares discriminant analysis
QC: quality control
RER: resveratrol-emodin-rhein
RRR: Rhei Radix Et Rhizoma
T: taurocholic acid sulfate
TCA cycle: tricarboxylic acid cycle
TCDCA: taurochenodeoxycholic acid
TDCA: taurodeoxycholic acid
THDCA: taurohyodeoxycholic acid
TU: trihydroxycholic acid
TUDCA: tauroursodeoxycholic acid
UCA: ursocholic acid
UDCA: ursodeoxycholic acid
YYD: Yiyi decoction
