# Supplementary material for: Integrated global and unique metabolic characteristics to reveal the intervention effect of Yiyi decoction on acute pancreatitis
Source: PLoS One. 2024 Nov 21;19(11):e0310689. doi: 10.1371/journal.pone.0310689 (PMC11581250; doi:10.1371/journal.pone.0310689)
Supplement: S1 Fig — (DOCX) [file pone.0310689.s001.docx]

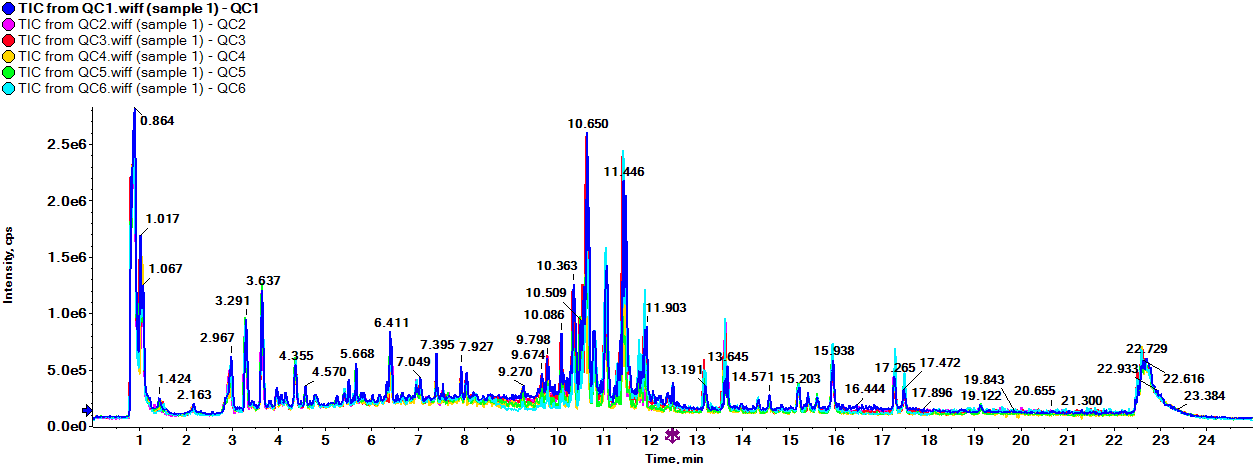


**(a)**

**
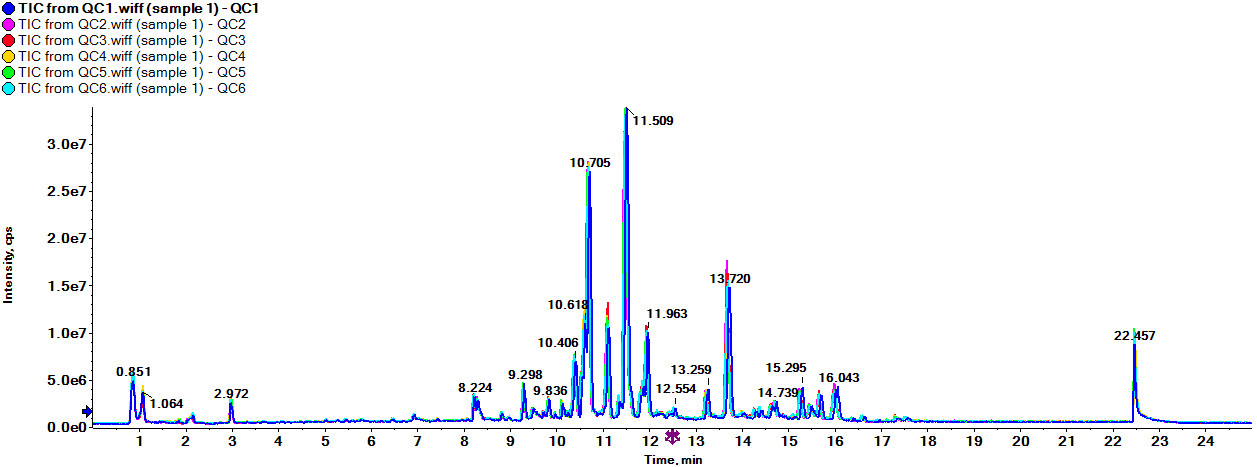
**

**(b)**

**Figure S1.** The overlapping total ion chromatograms (TICs) of QC samples in positive and negative modes. (a) Overlapping TICs in negative mode. (b) overlapping TICs in positive mode.
