# Supplementary material for: Integrated global and unique metabolic characteristics to reveal the intervention effect of Yiyi decoction on acute pancreatitis
Source: PLoS One. 2024 Nov 21;19(11):e0310689. doi: 10.1371/journal.pone.0310689 (PMC11581250; doi:10.1371/journal.pone.0310689)
Supplement: S2 Fig — (DOCX) [file pone.0310689.s002.docx]

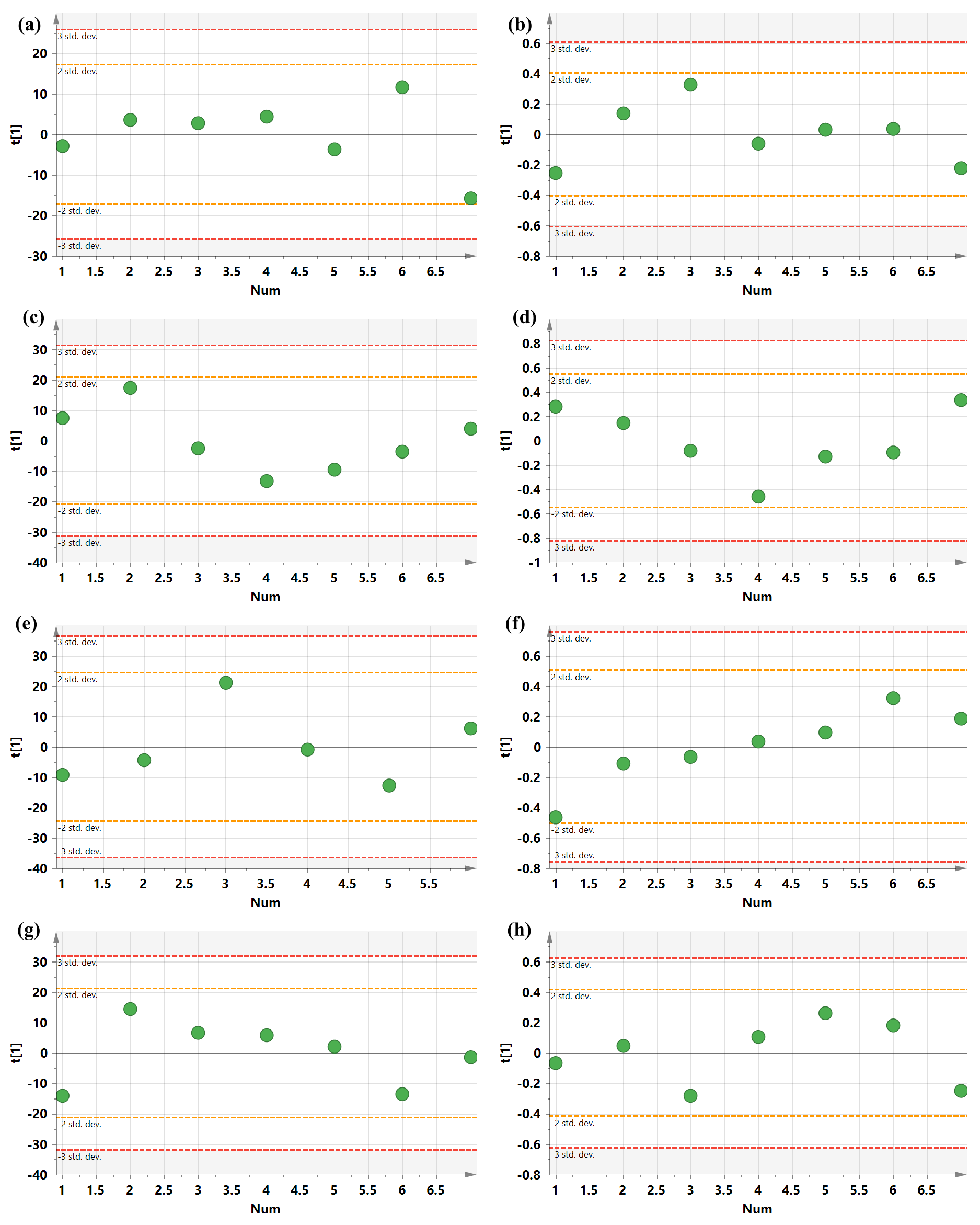


**Figure S2**. PCA-Class score plots in positive and negative modes. (a) Control group in negative mode; (b) Control group in positive mode; (c) Model group in negative mode; (d) Model group in positive mode; (e) YYD group in negative mode; (f) YYD group in positive mode; (g) RER group in negative mode; (h) RER group in positive mode. All of the samples were in the 2 std line.
