# Supplementary material for: Integrated global and unique metabolic characteristics to reveal the intervention effect of Yiyi decoction on acute pancreatitis
Source: PLoS One. 2024 Nov 21;19(11):e0310689. doi: 10.1371/journal.pone.0310689 (PMC11581250; doi:10.1371/journal.pone.0310689)
Supplement: S3 Fig — (DOCX) [file pone.0310689.s003.docx]

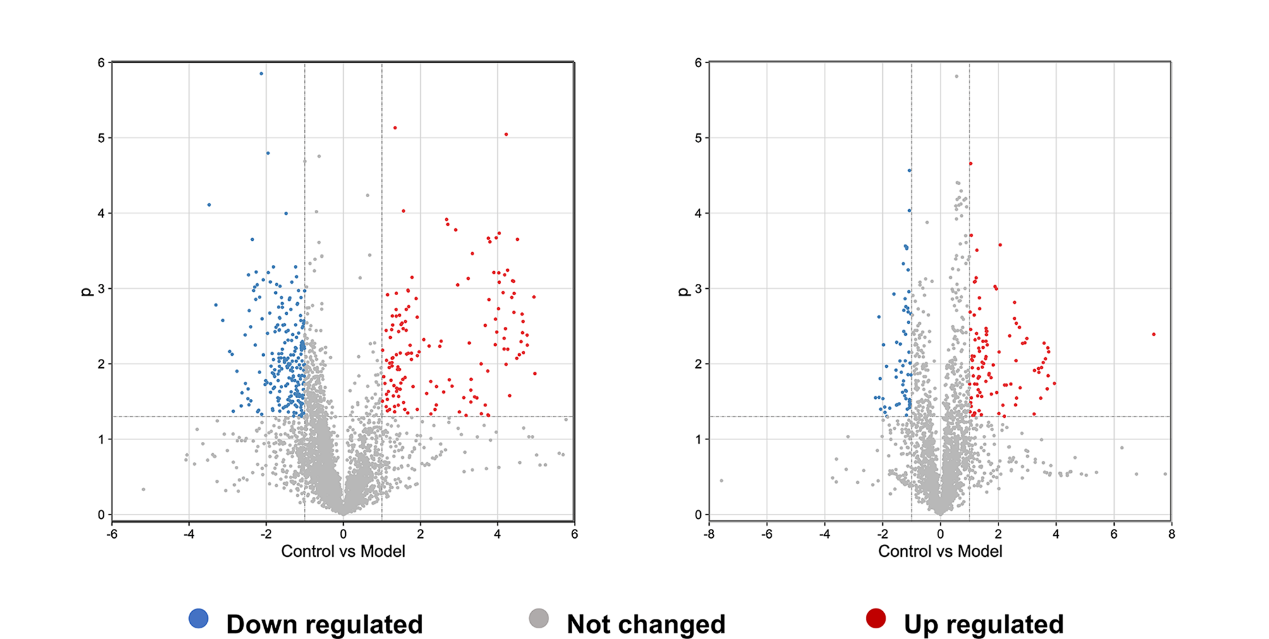


**Figure S3.** Volcano Plots of Control *vs* Model. (a) negative mode, (b) positive mode. ①Fold change (M/C) > 2 or < 0.5, and ②*P* < 0.05 would be considered significant. Significantly downregulated variables were colored blue while upregulated ones were colored red.
