# Supplementary material for: Integrated global and unique metabolic characteristics to reveal the intervention effect of Yiyi decoction on acute pancreatitis
Source: PLoS One. 2024 Nov 21;19(11):e0310689. doi: 10.1371/journal.pone.0310689 (PMC11581250; doi:10.1371/journal.pone.0310689)
Supplement: S4 Fig — (DOCX) [file pone.0310689.s004.docx]

|   TUDCA |   THDCA |
| --- | --- |
|   TDCA |   TCDCA |
|   T1-T4 |   UDCA |
|   DCA |   TU1/ TU2 |
|   UCA |   DU1/ DU2 |
|   3-oxo-CA |  |

**Figure S4**. Structures of 16 bile acids extracted in serum.
